# FM Dye Photo-Oxidation as a Tool for Monitoring Membrane Recycling in Inner Hair Cells

**DOI:** 10.1371/journal.pone.0088353

**Published:** 2014-02-05

**Authors:** Dirk Kamin, Natalia H. Revelo, Silvio O. Rizzoli

**Affiliations:** 1 Department of Neuro- and Sensory Physiology, University Medical Center Göttingen, Göttingen, Germany; 2 STED Microscopy of Synaptic Function, European Neuroscience Institute, Göttingen, Germany; 3 International Max Planck Research School Neurosciences, Göttingen, Germany; 4 Collaborative Research Center 889, University of Göttingen, Göttingen, Germany; 5 Cluster of Excellence Nanoscale Microscopy and Molecular Physiology of the Brain, University of Göttingen, Göttingen, Germany; University of Edinburgh, United Kingdom

## Abstract

Styryl (FM) dyes have been used for more than two decades to investigate exo- and endocytosis in conventional synapses. However, they are difficult to use in the inner hair cells of the auditory pathway (IHCs), as FM dyes appear to penetrate through mechanotransducer channels into the cytosol of IHCs, masking endocytotic uptake. To solve this problem we applied to IHCs the FM dye photo-oxidation technique, which renders the dyes into electron microscopy markers. Photo-oxidation allowed the unambiguous identification of labeled organelles, despite the presence of FM dye in the cytosol. This enabled us to describe the morphologies of several organelles that take up membrane in IHCs, both at rest and during stimulation. At rest, endosome-like organelles were detected in the region of the cuticular plate. Larger tubulo-cisternal organelles dominated the top and nuclear regions. Finally, the basal region, where the IHC active zones are located, contained few labeled organelles. Stimulation increased significantly membrane trafficking in the basal region, inducing the appearance of labeled vesicles and cistern-like organelles. The latter were replaced by small, synaptic-like vesicles during recovery after stimulation. In contrast, no changes in membrane trafficking were induced by stimulation in the cuticular plate region or in the top and nuclear regions. We conclude that synaptic vesicle recycling takes place mostly in the basal region of the IHCs. Other organelles participate in abundant constitutive membrane trafficking throughout the rest of the IHC volume.

## Introduction

Communication across chemical synapses relies on the release of neurotransmitter from synaptic vesicles, through vesicle fusion to the presynaptic plasma membrane (exocytosis). To ensure that sufficient vesicles are available, specialized mechanisms retrieve the vesicle components from the plasma membrane (endocytosis) and form new synaptic vesicles, completing the process known as synaptic vesicle recycling. Studies focusing on conventional synapses from both the central and peripheral nervous systems have revealed several different mechanisms of vesicle recycling. One such mechanism is kiss-and-run, in which the fusion pore of the vesicle closes rapidly after exocytosis. Alternatively, the fused vesicle may be retrieved by clathrin-mediated endocytosis (CME), either directly from the plasma membrane, or from membrane infoldings that form on the surface of the synapse after abundant exocytosis. The latter mechanism is known as bulk endocytosis [1], [2].

Much information on synaptic vesicle recycling has been obtained by studies using styryl dyes, and especially FM 1-43 [3]. This is a well-known membrane probe that is taken up during endocytosis and fluoresces brightly in membranes. It thus provides an overview of where membrane retrieval takes place. Stimulating the dye-loaded preparations causes the release of FM dye from the labeled organelles. This dye loss provides a direct measure of exocytosis kinetics. FM dyes have been applied to many different preparations, including frog, mouse or *Drosophila* neuromuscular junctions [3]–[5], cultured neuronal synapses (for example [6]), the calyx of Held [7], or bipolar cells of the goldfish retina [8].

FM dyes have not been as often applied to cells that lack synaptic boutons, and rely instead on somatic release sites (somatic active zones). These cells are found in the sensory pathways for sound, balance and taste. The best-studied example of this class of cells is the auditory inner hair cell (IHC), located in the mammalian organ of Corti. Stimulus intensity is transformed in IHCs into graded receptor potentials, whose strength determines the amount of neurotransmitter released. Synaptic release is continuous in IHCs, unlike in conventional synapses, where it only takes place upon the arrival of action potentials. The abundant IHC exocytosis relies on a specialized structure, the synaptic ribbon, to tether and concentrate the vesicles at active zones [9]–[13], and must also rely on efficient vesicle recycling mechanisms.

A few studies have followed these mechanisms by applying FM dyes to IHCs. Initial observations suggested that FM 1-43 uptake is most abundant at the apical pole of the cells [14]–[16]. The dye molecules afterwards reached structures inside the IHC, and revealed many organelles. These observations led to the hypothesis that IHC endocytosis occurs primarily in the apical region of the cell. The newly endocytosed vesicles may then passage through ER-like organelles or through the Golgi apparatus, before synaptic vesicles can be reformed. The following organelles have thus been implicated in vesicle recycling in IHCs, based mostly on FM dye studies: endosomes forming at the apex of the cells, near the cuticular plate [14], [15], [17], the endoplasmic reticulum [15], and the Golgi apparatus [16], [18]. In addition, two other types of organelles have been suggested to participate in this process, based on electron microscopy observations: tubular organelles from the apical, nuclear or basal regions [19]–[21], and membrane infoldings and round organelles derived from these infoldings, termed cisterns [18], [22].

However, it was later observed that FM molecules enter rapidly into the cells (seconds), and that they also inhibit mechanotransduction currents, leading to the hypothesis that most dye is not taken up by endocytosis, but permeates through mechanotransducer channels [23], [24] or other channel types, like ATP receptors [25]. This complicates the interpretation of FM dye imaging experiments in IHCs, since much of the fluorescence observed may not be related to endocytosis. An additional problem in cells with somatic active zones is that their synaptic vesicles are not isolated in presynaptic boutons, as in conventional synapses. Therefore, synaptic vesicle recycling shares the cellular volume with many other trafficking processes, which take place at the same time. This implies that at least some of the organelles mentioned in the previous paragraph may be involved in constitutive membrane trafficking, and not in synaptic vesicle recycling.

We explored here the possibility of studying membrane trafficking in IHCs using the technique of photo-oxidation electron microscopy, in which the FM dye taken up by the organelles is converted to a dark precipitate that is visible in electron microscopy. FM dye photo-oxidation has been used successfully in numerous conventional synapses, including the neuromuscular junctions of frog, fruit fly, locust, zebrafish, mouse and *Caenorhabditis elegans* [26]–[30], rat hippocampal cultured neurons [31], [32], and rodent, fruit fly or cricket brain slices or whole brains [7], [30], [33]. We have adapted the technique for IHCs. The problems encountered with FM dye imaging in IHCs were no longer relevant for photo-oxidation experiments. Photo-oxidation revealed only the endocytotic organelles. No labeled organelles were detected in conditions in which endocytosis was inhibited by low-temperature incubation, although abundant FM dye entry into the cytosol was still observed. This technique thus allowed us to observe endocytosis in IHCs without any interference from FM dye entry through mechanotransducer channels. We found that synaptic vesicle recycling took place in the basal region of the cell, where the active zones are located. Abundant constitutive membrane trafficking took place in the rest of the cell, relying on endosome-like organelles found in the apical region of the cell, and on tubulo-cisternal organelles from the top and nuclear regions.

## Results

### Fluorescence imaging of FM 1-43 does not report endocytosis accurately in IHCs

As indicated in the Introduction, it is still debated whether FM dyes can be used as reliable reporters of endocytosis in IHCs. To verify this, we incubated IHCs with FM 1-43, the most widely used styryl dye, and verified the IHC labeling by fluorescence microscopy.

**Figure 1:**
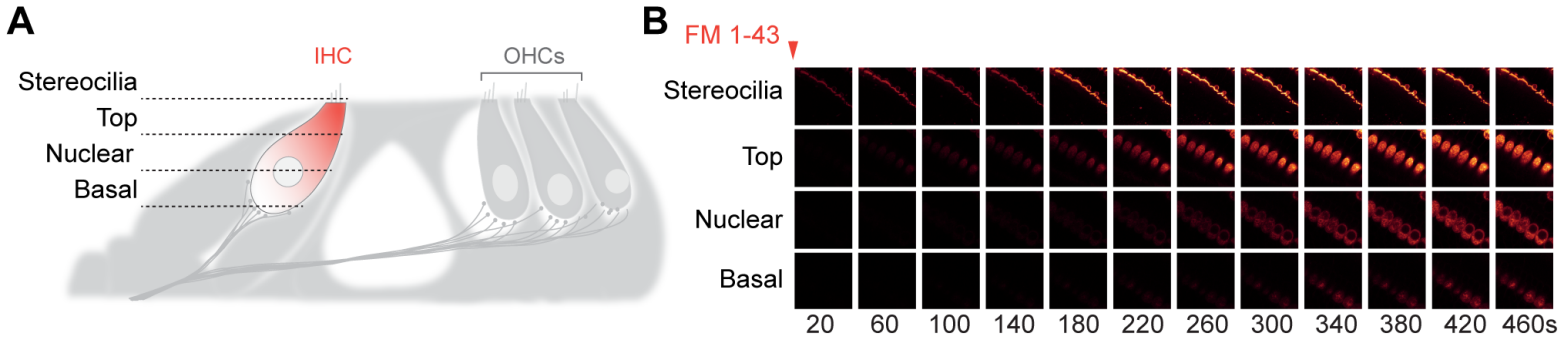
FM 1-43 penetrates rapidly into IHCs. (**A**) Scheme of the organ of Corti, indicating the areas of the IHCs that were imaged. (**B**) FM 1-43 (10 µM) labeling of IHCs was imaged at room temperature at four cellular levels: stereocilia bundle, top, nuclear and basal. FM 1-43 reaches the cytoplasm, diffusing in an apex-to-base fashion (right panel). This observation is typical for 16 independent experiments.

The apical turn of the organ of Corti (Fig. 1A), dissected from wild-type mice at ages between P14 and P18, was incubated for 60 seconds at room temperature in a bathing solution containing 10 µM FM 1-43. The dye entered the IHCs rapidly, starting from the top and diffusing towards the basal level, in a fashion that is difficult to reconcile with endocytosis (Fig. 1B), but is fully compatible with FM 1-43 penetrating into the cells through the mechanotransducer channels.

It has been suggested in the past that chemical channel blockers prevent the entry of the dye into the cytosol, and thus enable the investigation of IHC endocytosis by FM 1-43 imaging. One such channel blocker is suramin, an antagonist of ionotropic purinergic (P2X) receptors, which was shown to reduce FM dye uptake in chick hair cells [25]. Suramin did reduce FM 1-43 fluorescence in our experiments (Fig. S1A). However, it does not appear to do so by inhibiting FM 1-43 penetration through the channels. The negative charges of suramin directly interact with the two positive charges of FM 1-43 (Fig. S1B; see also Fig. S1E). This interaction reduces FM 1-43 fluorescence even on the external leaflets of the plasma membranes of cultured cells (Fig. S1C and D). Therefore, suramin cannot be used to enable FM 1-43 imaging in IHCs.

### FM 1-43 analogues, as well as unrelated fluorescent markers, fail to report endocytosis in IHCs

**Figure 2:**
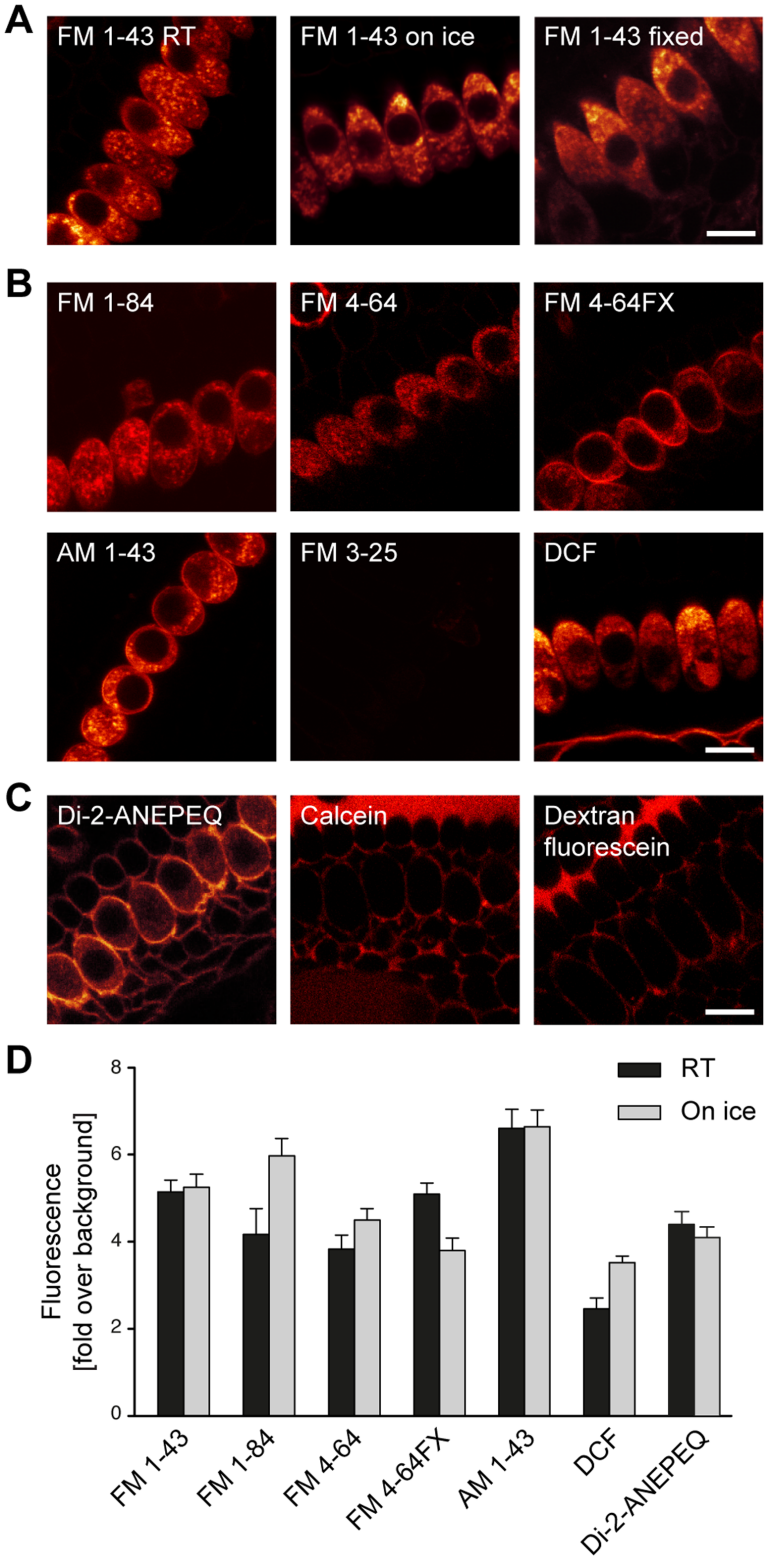
Most commercial fluorescent markers fail in reporting endocytosis in IHCs. (**A**) FM 1-43 labels IHCs strongly in conditions where endocytosis is strongly reduced by low temperature (on ice, middle panel) or abolished by fixation (right panel). The labeling pattern is identical to that obtained at room temperature (RT, left panel). (**B**) Styryl dyes from the FM family (FM 1-84, FM 4-64, FM 4-64FX, AM 1-43), ranging in sizes between 450 and 470 Da, permeated IHCs in a similar fashion to FM 1-43 at room temperature. In contrast, the larger FM 3-25 (843 Da) did not penetrate into the tissue, producing a very faint labeling. The membrane-binding dye 5-dodecanoylaminofluorescein (DCF, 530 Da) had a similar effect. (**C**) An additional membrane dye, Di-2-ANEPEQ (549 Da), labeled IHCs in the same fashion as FM 1-43. Soluble compounds, such as calcein (623 Da) and 3000 Da Dextran coupled to fluorescein, labeled the intercellular spaces throughout the organ of Corti, but were not taken up efficiently by the cells. All dyes were used at a concentration of 10 µM, with the exception of Di-2-ANEPEQ, which was used at 100 µM, and DCF, used at 180 µM. Scale bars 10 µm. (**D**) Analysis of labeling intensity for all dyes that appeared to penetrate into the IHCs. The fluorescence intensity of the cells (excluding the nucleus) was normalized to the background intensity, and is expressed as fold over background. The black bars show results from cells incubated with the different dyes at room temperature. The gray bars show cells incubated on ice, at 2–4°C. The following numbers of IHCs were analyzed. FM 1-43: 26 at room temperature, 19 on ice; FM 1-84: 7, 27; FM 4-64: 13, 23; FM 4-64FX: 8, 20; AM 1-43: 20, 16; DCF: 8, 15; Di-2-ANEPEQ: 30, 35.

To verify further whether the strong and rapid FM 1-43 labeling of IHCs was related to endocytosis, we incubated the cells with FM 1-43 on ice (2–4°C), a temperature at which endocytosis is inhibited. The FM 1-43 labeling was virtually unmodified (Fig. 2A). The labeling pattern was strikingly similar to that of fixed cells, in which the FM dye penetrates through pores in the plasma membrane (Fig. 2A; see also Fig. S2 for a discussion of the FM dye labeling of fixed cells). These results confirm that a pathway unrelated to endocytotic dye uptake is responsible for most FM 1-43 labeling in IHCs.

It was nevertheless still possible that other FM dyes, with different sizes and different membrane-binding properties, report endocytosis more accurately. We therefore tested several commercially available FM dye analogues: the red-shifted FM 4-64, the more hydrophobic FM 1-84 and FM 3-25, as well as two analogues that contain a lysine residue, AM 1-43 and FM 4-64FX (Fig. 2B). We also tested two additional lipid dyes, 5-Dodecanoylaminofluorescein and Di-2-ANEPEQ (also known as JPW1114, Fig. 2B–C). With the exception of the highly hydrophobic FM 3-25 [24], which did not reach the IHCs within the organ of Corti on the time scale of our experiments (a few minutes), all of the dyes resulted in rapid and abundant labeling, similar to that of FM 1-43. Additionally, these dyes also labeled IHCs kept at low temperature in a similar fashion (Fig. 2D, example images in Fig. S3). This observation confirms that they, just as FM 1-43, report a labeling process that is not related to endocytosis.

Soluble molecules can also, in principle, be used as tracers for endocytosis. We therefore tested calcein, a polyanionic derivative of fluorescein, and a 3000-Da variant of Dextran coupled to fluorescein. They both filled intercellular spaces but were taken up with far too limited efficiency to serve as markers for endocytosis (Fig. 2C).

These observations forced us to conclude that several conventional fluorescent markers, which have been used successfully in the past to report membrane trafficking in neurons and other cell types, are difficult to apply to IHCs.

### FM 1-43 photo-oxidation successfully reports endocytosis in IHCs

FM dyes have been used in the past to investigate the morphology of recycling organelles from conventional synapses in electron microscopy, through a technique termed FM dye photo-oxidation [26]. The preparations are labeled with FM dyes and are then fixed and illuminated in presence of di-amino-benzidine (DAB), a compound that forms an insoluble precipitate upon oxidation. Illumination of the area of interest degrades the dye molecules, giving rise to reactive oxygen species that oxidize DAB. The electron-dense DAB precipitate is subsequently identified in electron microscopy (see protocol in [34]; see also references [7] and [26]–[33]).

This technique has not been yet used in cells with somatic active zones, such as the IHCs. A related method, also based on DAB precipitation, has been used in a seminal study of IHC membrane traffic [18]: horseradish peroxidase (HRP) uptake, followed by DAB precipitation in the HRP-labeled organelles. However, only few labeled vesicles were detected, even after 30 minutes of HRP incubation *in vivo*, at the physiological temperature of the animals [18]. The reason for this may be that HRP, a 44 kDa protein, is not taken up by recycling vesicles as efficiently as the much smaller FM dyes [30]. Additionally, HRP requires long time intervals to diffuse into tissues. Confirming this reasoning, we were unable to note efficient HRP uptake into IHCs when incubating the organs of Corti for 10–20 minutes at room temperature (Fig. S4). Since longer incubations are not compatible with the investigation of relatively rapid processes such as synaptic vesicle endocytosis and recycling, we did not explore this issue further, preferring to focus on FM dyes, which partition into tissues within seconds.

**Figure 3:**
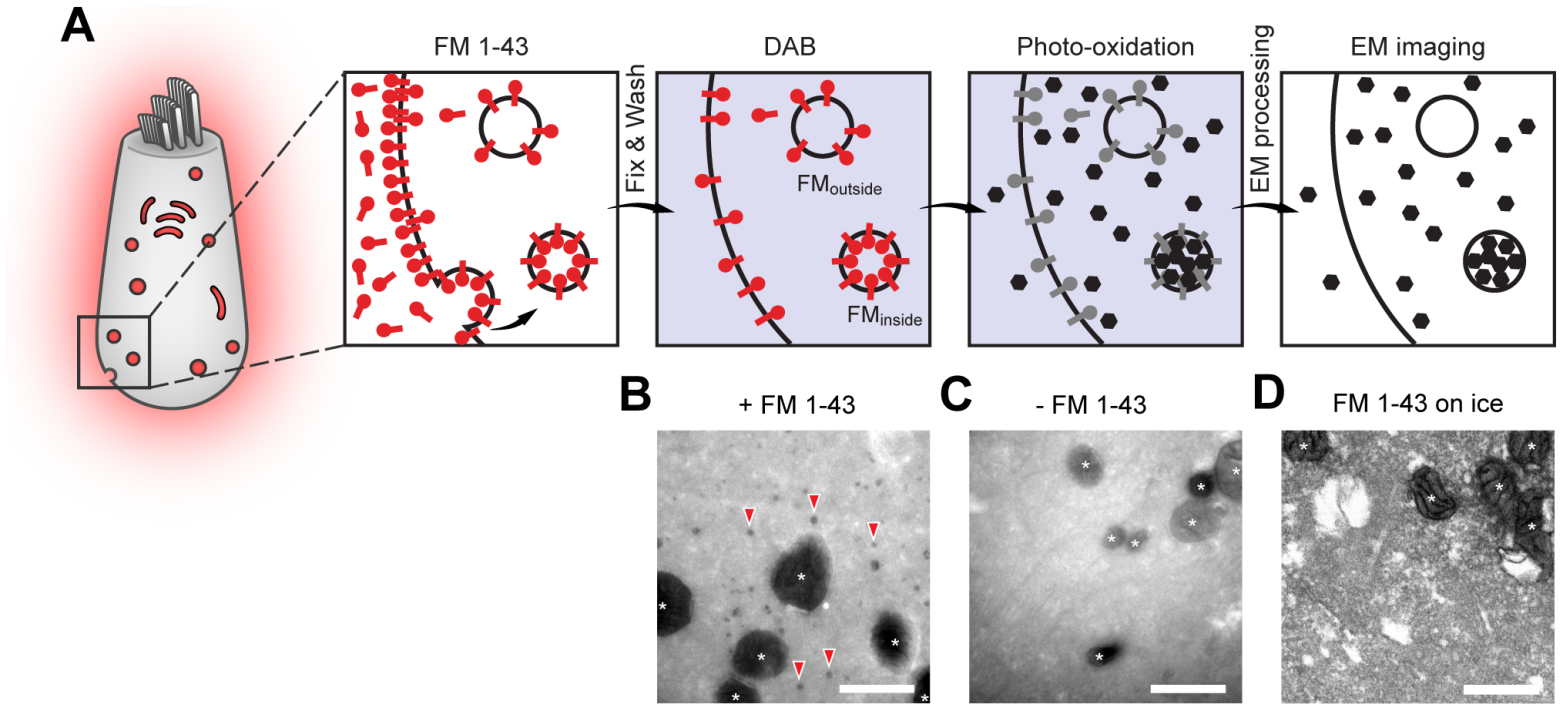
FM 1-43 can be used as an endocytosis tracer using photo-oxidation electron microscopy. (**A**) Experimental outline: IHCs incubated with FM 1-43 take up the dye (red symbols) in endocytotic organelles, but also in the cytosol, as a result of dye penetration through mechanotransducer channels. The dye in the cytosol attaches itself to the external (cytosolic) leaflets of the organelle membranes. After fixation and incubation with DAB (indicated as the blue background), the cells are illuminated with blue light; the FM molecules bleach (gray symbols) and emit reactive oxygen species that cause DAB precipitation (represented as black symbols). The DAB precipitate diffuses in the cytosol, unless it is trapped within the organelles. Therefore, endocytotic organelles are seen as black objects, while the FM dye in the cytosol only causes a general darkening of the cell volume. (**B**) In presence of the dye numerous dark organelles appear (some examples are indicated by red arrowheads). (**C**) In the absence of the dye only mitochondria are visibly labeled. Mitochondria are labeled by photo-oxidation by mechanisms independent of the addition of fluorescent markers, and are indicated by white asterisks. (**D**) Apart from mitochondria, no labeled organelles were found in cells incubated with FM 1-43 at low temperature (2–4°C), confirming that dye photo-oxidation selectively reports endocytotic events – despite the fact that FM 1-43 penetrates into the IHC cytosol at low temperature. The image is over-exposed, to increase the contrast as much as possible, which should allow the detection of any labeled organelles. Nevertheless, none are present, other than mitochondria. Scale bars, 500 nm.

Previous observations with FM 1-43 photo-oxidation [30], and with photo-oxidation of cytosolic dyes (such as ReAsH bound to soluble proteins), led us to the conclusion that the procedure induces a dark, solid precipitate only for the dye trapped inside organelles. Free dye in the cytosol, which binds the extracellular leaflet of the organelles, leads to the formation of diffuse DAB precipitate, which renders the entire cytoplasm electron-denser, but does not impede the identification of trafficking organelles. Thus, although FM dye penetration through mechanotransducer channels did not allow us to describe endocytosis through fluorescence imaging in IHCs, it should have been irrelevant when investigating endocytotic organelles by photo-oxidation electron microscopy (Fig. 3A).

We photo-oxidized IHCs that were incubated at 37°C with FM 1-43 (5 µM, 60 seconds). In presence of the dye, abundant labeled organelles were observed (Fig. 3B). IHCs treated identically, but in the absence of FM dye, lacked such organelles (Fig. 3C). The DAB precipitate also accumulated in mitochondria, due to oxidation caused by mitochondrial enzymes and by mitochondrial auto-fluorescence, unrelated to FM dyes or endocytosis [30], [35] (large dark organelles in Fig. 3B–D). As expected, this phenomenon took place also in IHCs not exposed to FM dye: the mitochondria were the only electron-dense organelles in these cells (Fig. 3C).

We also photo-oxidized IHCs incubated with FM 1-43 at low temperature (on ice, at 2–4°C). While these cells show abundant fluorescence labeling (Fig. 2A), no organelles labeled by photo-oxidation were detected (Fig. 3D). This confirms our hypothesis that only the dye taken up by endocytosis leads to photo-oxidized organelles (Fig. 3A). These experiments thus suggest that FM 1-43 photo-oxidation can be used to investigate membrane trafficking in IHCs.

### Membrane trafficking at the base of IHCs is most relevant to synaptic activity

As suggested in the Introduction, a difficulty in understanding membrane trafficking in IHCs is that several organelles have been suggested to take place in this process: the ER, the Golgi apparatus, apical endosome-like organelles, tubular organelles from all regions of the cell, as well as vesicles and cisterns found in the basal region [14]–[22].

To determine which of these organelles are relevant to synaptic vesicle recycling, we decided to compare resting IHCs, in which only constitutive membrane trafficking takes place, with stimulated (depolarized) IHCs, in which both constitutive membrane trafficking and synaptic vesicle recycling take place. We hypothesized that any differences in organelle abundance or morphology that are induced by stimulation are probably related to synaptic vesicle recycling. In contrast, organelles that do not respond to stimulation are unlikely to participate in synaptic vesicle recycling. Finally, to determine the fate of the different endocytotic organelles, we also investigated the IHCs during recovery after stimulation.

The following conditions were employed: resting (in Hanks balanced salt solution, HBSS, with 5 mM K^+^, without Ca^2+^, for 60 seconds), stimulation (HBSS with 65 mM K^+^ and 2 mM Ca^2+^, again for 60 seconds), and recovery (stimulation in presence of the dye followed by wash-off and incubation in HBSS with 2 mM Ca^2+^ for a 5-minute or a 30-minute recovery period).

**Figure 4:**
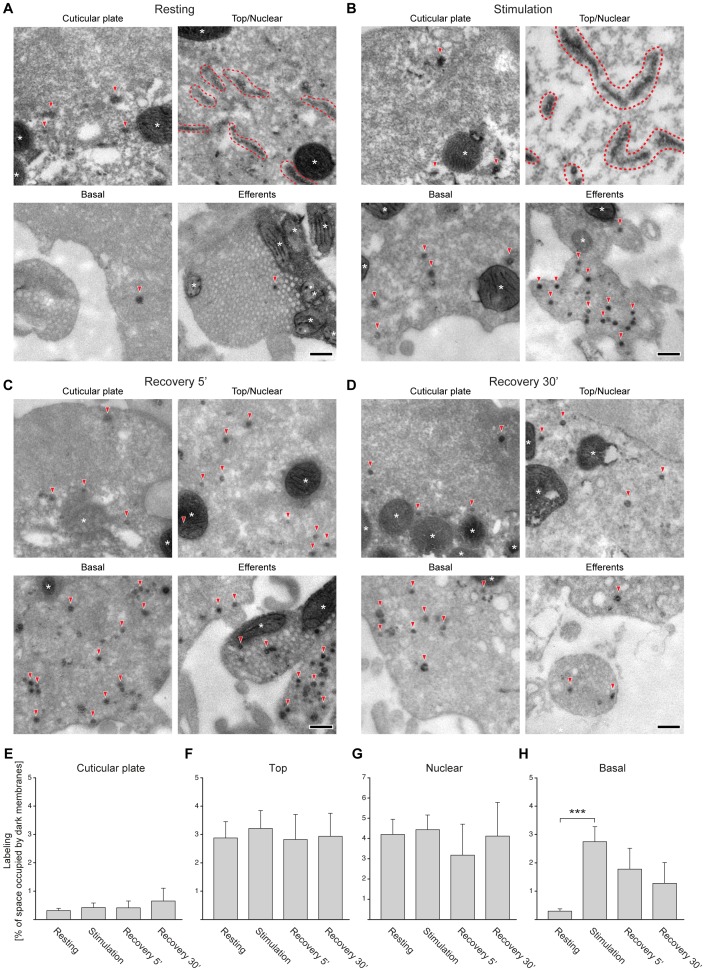
FM 1-43 photo-oxidation suggests that synaptic vesicles recycle at the base of IHCs. (**A**)–(**D**) The electron micrographs show organelles containing the photo-oxidation product (DAB precipitate) at rest (**A**), during stimulation (**B**), or after a 5-minute (**C**) or 30-minute (**D**) recovery period. Some typical organelles are indicated by red arrowheads. Large tubular organelles are indicated by the dashed red traces. Mitochondria are indicated by white asterisks. Note that the basal region contains few labeled organelles at rest; their number increases after stimulation. Note also that the tubular organelles from the top and nuclear regions, found both at rest and during stimulation, disappear during the recovery periods. Scale bars, 200 nm. (**E**–**H**) Analysis of organelle labeling under the different experimental conditions. The percentage of the cell volume occupied by labeled organelles was measured in four different cellular regions: cuticular plate (**E**), top (**F**), nuclear (**G**) and basal (**H**). The graphs show averages performed for electron micrographs from 14 (resting), 12 (stimulation), 4 (5 minute recovery) and 4 (30 minute recovery) independent experiments. We used one-way ANOVA tests to check whether stimulation produced any changes in the different cellular regions. No significant differences were found for the cuticular plate region (**E**), for the top region (**F**), and for the nuclear region (**G**); all *P* values were higher than 0.05. In contrast, the ANOVA test revealed significant differences for the basal region (**H**); *P*<0.05. A *post hoc* Bonferroni test indicated that stimulation increases the amount of label significantly in this region, compared to the resting control (*P*<0.001).

All IHCs, under all conditions, contained labeled organelles. Examples of organelles labeled under resting, stimulation and recovery conditions are shown in Fig. 4A–D. An overview of membrane trafficking throughout the IHCs allowed us to define three broad areas, according to the type of labeled organelles they contained. First, the cuticular plate area, which was characterized by the presence of only a few labeled organelles. These were round, endosome-like in morphology, as described in the past [14], [15], [17], and did not appear to change in response to stimulation.

Second, the top and nuclear areas of the cells, which were dominated by large tubular organelles, typically surpassing 500 nm in length, and with a breadth of ∼100 nm or less. These organelles were visible both at rest and during stimulation (Fig. 4A–B). No change in the abundance or morphology of tubular organelles seemed to take place upon stimulation. They appeared to divide into smaller organelles during the recovery period (Fig. 4C–D).

Third, the base of the IHC contained few labeled organelles under resting conditions. This changed upon stimulation, with the base of the cell becoming populated with small labeled vesicles and cisterns. The latter (defined according to [22]) are round or oval in shape, of a few hundred nanometers in diameter (but typically less than 500 nm), and with a breadth that is close to their length (i.e., oval or circular in shape). They also seemed to break up into smaller organelles during recovery.

Overall, these observations suggest that the membrane turnover at the base of the IHC may respond most strongly to stimulation. A quantitative analysis of the total volume occupied by the endocytotic organelles confirmed this (Fig. 4E–H): stimulation left the organelles in the cuticular plate, top and nuclear areas unaffected, but strongly enhanced endocytosis at the cell base. We therefore conclude that membrane trafficking at the base of the cell is the only pathway likely to be involved in synaptic vesicle recycling. The other membrane pathways are independent of stimulation, and therefore are independent of synaptic vesicle exocytosis.

### 3D reconstructions confirm that membrane trafficking is abundant in all areas of the IHCs, both at rest and during stimulation

The results shown in Fig. 4 were obtained by sampling individual IHC sections. This procedure generally provides similar results to in-depth studies of 3D reconstructions [28]–[30]. Nevertheless, we decided to confirm our findings by reconstructing large portions of IHCs treated as in Fig. 4 (resting, stimulated, and allowed to recover after stimulation for 5 or 30 minutes).

**Figure 5:**
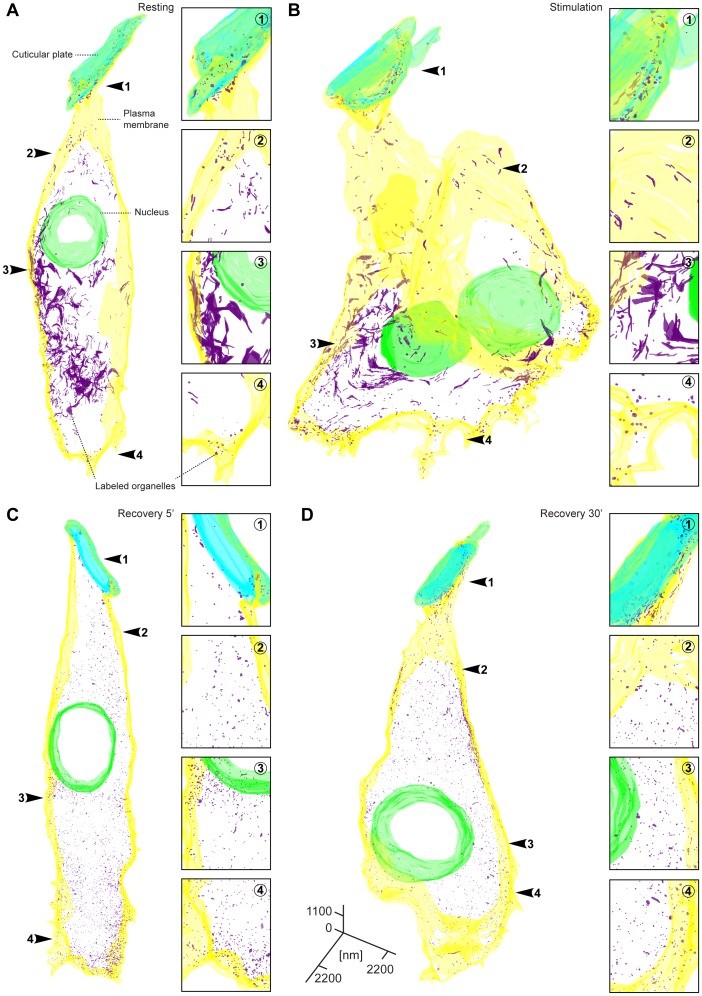
3D reconstructions confirm the observations derived from single IHC sections. 3D reconstructions of resting (**A**), stimulated (**B**) and recovered IHCs (**C** and **D**). Endocytotic organelles are shown in purple. Note the presence of tubular organelles both before and after stimulation. Most organelles, including the tubular ones, are replaced by small vesicles during the recovery periods. Insets show magnified regions from the four different cell region (cuticular plate, top, nuclear and basal regions). Note the increased number of endosome-like organelles at the base of the cell after stimulation and during recovery.

The membranes of organelles containing DAB precipitate were manually drawn onto approximately 2000 electron micrographs, obtained from 20 serial longitudinal sections of single IHCs (section thickness 100 nm). We thus reconstructed volumes of approximately 1000 µm^3^, spreading from the cuticular plate to the base of the cell (Fig. 5A–D). The 3D reconstructions confirmed our previous observations (Fig. 4). Tubular organelles found in the top and nuclear areas contained the large majority of endocytotic label, both at rest and during stimulation (Fig. 5A, B). Smaller organelles, such as cisterns, were poorly represented at rest (Fig. 5A), and their number increased upon stimulation (Fig. 5B).

During the recovery period most of the labeled organelles, with the exception of a proportion of the tubules, were replaced by small vesicles, of less than 100 nm in diameter (Fig. 5C–D). This suggests that both the cisterns from the basal region and the tubules from the top and nuclear regions can be processed into smaller vesicles. The 3D reconstructions also allowed us to describe the structure of the tubules in more detail (Fig. S5). Although they appear to be long tubules in single sections, their true structure was revealed by 3D reconstructions: they contain both elongated and flattened regions, being sheet-like in appearance, rather than tube-like.

### The small vesicles forming in the top and nuclear areas are larger than synaptic vesicles

**Figure 6:**
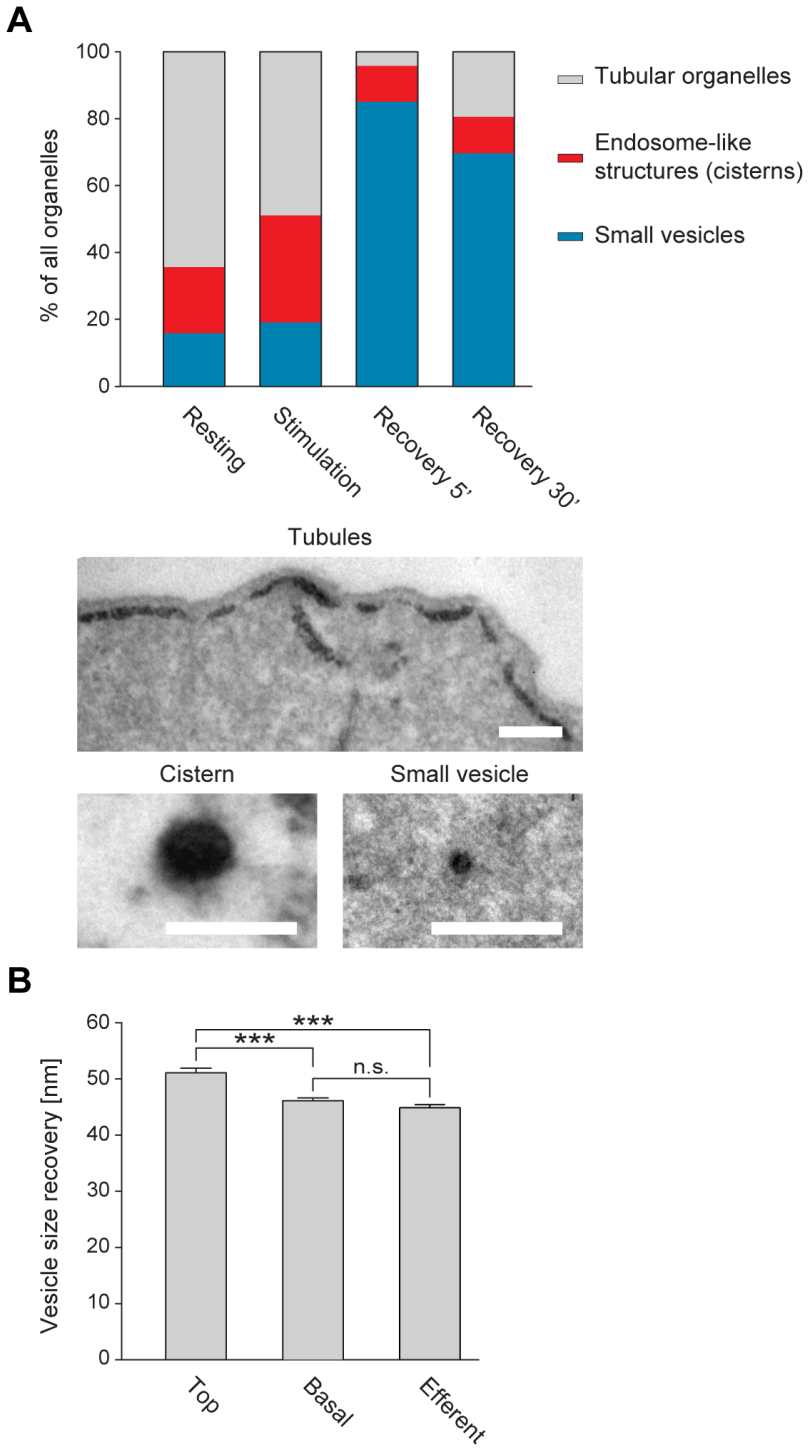
The vesicles forming during recovery in the top region of the IHCs are significantly larger than synaptic vesicles. (A) Analysis of the organelle numbers from the 3D reconstructions presented in Fig. 5. Organelles containing the photo-oxidation product were classified in three categories: tubules (large elongated or flattened organelles), cisterns (round or elliptic organelles) and small vesicles. Tubules dominate at rest. Cisterns are induced to form during stimulation. Almost all organelles were processed to small vesicles during recovery. Images show representative organelles for each of the categories. Scale bar, 200 nm. (B) Quantification of the size of small vesicles (<120 nm) at the top and the base of the cell, after a 5-minute recovery. The latter are significantly smaller, and very similar to *bona fide* synaptic vesicles from the efferent boutons. The bars show means ± SEM, from 260, 690 and 219 vesicles (top, base and efferent, respectively). A one-way ANOVA test indicated that the vesicle populations were significantly different. A *post hoc* Bonferroni test revealed that the vesicles at the top of the IHCs were significantly larger than those in the basal region, and than those from the efferent boutons (*P*<0.0001). The latter were not significantly different from each other (*P*>0.05). The analysis included all small vesicles found in the relevant IHC regions in the 3D reconstructions presented in Fig. 5. The data points were sufficiently numerous to allow us to verify that the distributions were bell-shaped (using histograms of vesicle size). The variance was similar between the data groups: approximately 72, 190 and 143 units for top, base and efferent, respectively. The graphs show the entire data set we obtained in the 3D reconstructions; each 3D reconstruction is derived from one representative experiment.

A quantitative determination of the numbers of labeled organelles confirmed the impressions given by the 3D reconstructions: stimulation increased the number of cisterns, and during recovery most of the organelles turned into small vesicles (Fig. 6A).

Superficially, this would appear to imply that the large, tubular organelles are implied in synaptic vesicle recycling, since they also can be processed into small vesicles, just as the cisterns. We investigated this issue by measuring the size of the small vesicles forming after recovery in the top and nuclear areas, versus that of vesicles forming at the base of the cell (Fig. 6B). The former averaged over 50 nm in diameter, significantly larger than the vesicles forming at the base of the cell. These were close to ∼45 nm, and were indistinguishable from *bona fide* synaptic vesicles that were measured in efferent presynaptic boutons underneath the IHCs (conventional presynaptic terminals that should recycle synaptic vesicles by means common to most such boutons [1]).

We conclude that although small vesicles form both in the top and basal regions of IHCs, only the ones forming at the base of the cell are similar to *bona fide* synaptic vesicles. The top ones are larger than synaptic vesicle. This observation is in agreement with the finding that stimulation leaves this region unaffected (Fig. 4), and again suggests that membrane trafficking at the base of the IHCs is more relevant for synaptic vesicle recycling than trafficking elsewhere.

## Discussion

### A model of synaptic vesicle recycling in IHCs

We have used here FM dye photo-oxidation and electron microscopy to analyze endocytotic intermediates and their processing into small vesicles throughout IHCs. These findings allow us to generate the following hypothetical model of membrane trafficking in IHCs. First, constitutive membrane trafficking is abundant in IHCs. It takes place both at rest and during stimulation, throughout the cell. Much of the endocytosed material reaches tubular organelles in the top and nuclear areas of the cell. Within minutes, the tubules may be processed to small vesicles that are nevertheless significantly larger than synaptic vesicles. The nature of the tubules is still unclear, although they are probably related to early endosomes that recycle membrane on short temporal scales in a variety of cells (Revelo et al., unpublished observations).

Second, synaptic vesicle recycling takes place after stimulation selectively at the base of the cell. Synaptic vesicles presumably exocytose in the vicinity of the somatic active zones, and membrane is taken up locally in the form of cisterns. The cisterns are later turned into small vesicles, in a process that may be similar to the bulk endocytosis described in conventional synapses (see below).

These findings are in agreement with several electron microscopy studies of IHCs. For example, an early study using HRP as endocytosis tracer found a variety of labeled organelles within the cells, ranging from small coated and uncoated vesicles to large endosome-like vesicles, tubules and even Golgi stacks [18]. The observation of labeled Golgi stacks, which we could not reproduce in our experiments, is likely due to the much longer incubation time in presence of the endocytotic marker used in this study: 30 minutes, rather than 1 minute in our study. The longer incubation time likely allowed the observation of retrograde traffic to the Golgi apparatus through a constitutive pathway [18].

Our results are also in agreement with earlier observations of tubules in IHCs, although some of the previous studies reported IHC tubules that bore ribosomes, and which may represent ER membranes [19], [20]. Finally, our findings are compatible with electron tomograms of active zones in frog saccular hair cells [22], which demonstrated that stimulation triggers the formation of plasma membrane infoldings and cisterns, which are later processed into new synaptic vesicles.

### Similarities to other synaptic vesicle recycling pathways

The pathway employed by IHCs to recycle vesicles that we describe here is similar to that of another well-studied ribbon synapse, from the bipolar neurons in the retina. These neurons have a large nerve terminal that houses hundreds of thousands of synaptic vesicles, segregated from the cell body by an axon of a few tens of micrometers. The massive membrane recycling that follows neurotransmitter release in these synapses takes place through membrane infoldings that pinch off, forming cisterna-like structures [36] that are later divided into synaptic vesicles [37].

The formation of cisterns which later give rise to synaptic vesicles recalls a model of synaptic vesicle recycling from conventional synapses, termed bulk endocytosis. Bulk endocytosis is used by conventional synapses, including small CNS boutons and neuromuscular junctions, when stimulation overruns other mechanisms such as kiss-and-run and clathrin-mediated endocytosis. Under mild stimulation, many conventional synapses retrieve their vesicles directly from the plasma membrane by the latter mechanisms, without formation of other endocytotic intermediates [2]. After stronger stimulation, however, exocytosis increases to levels that cannot be dealt with by these mechanisms. The exocytosed material accumulates on the plasma membrane, and the synapses respond by forming membrane infoldings. The infoldings may break off and form cisterns, from which then clathrin and its cofactors slowly detach single vesicles [2], [38], [39].

Bulk endocytosis may be a regularly used mechanism in IHC synapses. Synaptic release is here substantially higher than in conventional synapses [13], and thus may require the membrane trafficking mechanisms employed by conventional synapses exclusively under strong stimulation.

One apparent difficulty is that the formation of membrane infoldings, which are then processed into synaptic vesicles, implies relatively slow vesicle reformation. Kiss-and-run, and possibly even clathrin-mediated endocytosis directly from the plasma membrane, would be faster. However, the lower speed of this endocytosis mechanism is probably not limiting for the IHCs: they contain a large reserve pool of vesicles, which will replenish the ribbons and will thus ensure that synaptic release is not impaired. These vesicles are found throughout the IHC volume [40], [41], and are probably highly mobile, as are those in other cells with ribbon synapses [42], which will enable them to reach the ribbons efficiently.

Nevertheless, we would also like to point out that, as for conventional synapses, it is possible that membrane is retrieved by clathrin-coated vesicles directly from the plasma membrane, rather than from cisterns, after moderate IHC activity (see for example [18], [22]).

### Technical conclusion: although FM dyes imaging is not efficient in IHCs, their photo-oxidation enables the investigation of membrane trafficking

Our study demonstrates that FM dye photo-oxidation can be used to reveal endocytotic organelles in IHCs, although the dyes do not report endocytosis with sufficient accuracy in fluorescence imaging experiments. Two elements contribute to this. First, the fact that dye molecules are present in the cytosol poses no problems in photo-oxidation. Second, the high resolution of electron microscopy allows the accurate description of the morphologies of the different organelles.

Our experiments thus enlarge the number of preparations in which FM dye photo-oxidation has been used successfully (see references [26]–[33]). To our knowledge, FM dye photo-oxidation is typically used only when FM dye imaging is successful. Our data suggest that this does not need to be the case. Therefore, FM dye photo-oxidation should have a larger palette of applications than FM dye imaging, which remains to be exploited.

## Methods

### Ethics Statement

Mice were handled according to the specifications of the University of Göttingen and the State of Lower Saxony (Landesamt für Verbraucherschutz, LAVES, Braunschweig, Germany). The procedures were approved by the University of Göttingen Board for Animal Welfare and the Animal Welfare Office of the state of Lower Saxony.

### Animals

Mice (*Mus musculus*) were obtained from the animal facility of the University Medical Center Göttingen or from Charles River (Sulzfeld, Germany). Male or female wild-type mice from the substrains C57BL/6N or C57BL/6J at ages between postnatal days 14–18, were anesthetized with CO_2_ and sacrificed by decapitation for later dissection of the organs of Corti.

### Testing fluorescent dyes in living IHCs

The apical turn of the organ of Corti was dissected and directly placed in an imaging chamber filled with Hank's Balanced Salt Solution without calcium (HBSS without Ca^2+^) containing (in mM): 5.36 KCl, 141.7 NaCl, 1 MgCl_2_, 0.5 MgSO_4_, 10 HEPES, 3.4 L-Glutamine, and 6.9 D-Glucose, pH 7.4. FM 1-43, FM 1-84, FM 4-64, FM 3-25, and AM 1-43 were purchased from Biotium (Hayward, CA, USA). FM 4-64FX, Di-2-ANEPEQ (JPW1114), calcein, 5-docecanoylaminofluorescein and 3000 Da Dextran-fluorescein were purchased from Life Technologies GmbH (Darmstadt, Germany). Time-lapse z-stack series along the IHC longitudinal axis were obtained using a Leica SP2 upright confocal microscope equipped with a water immersion objective (63×, 0.9 NA, HCX APO L U-V-I) (Leica Microsystems GmbH, Wetzlar, Germany). FM dyes, 5-docecanoylaminofluorescein and Di-2-ANEPEQ were excited with the 488 nm line of an argon laser and their emission was detected in the range of 500–700 nm by a photomultiplier tube (PMT). Due to their high fluorescence in solution, which prohibited single-photon microscopy, calcein and Dextran fluorescein were excited at 800 nm with a two-photon Ti:Sapphire laser (1.0 W output power, Verdi-V8 femtosecond diode-pumped laser, pulsed by Mira Optima 900-F; Coherent, Santa Clara, USA). Emission was detected in the range of 500–660 nm with a PMT. For labeling at low temperature, the organ of Corti was pre-incubated for 5 minutes in a plastic plate containing ice-cold HBSS without Ca^2+^, followed by incubation in HBSS without Ca^2+^ + 10 µM FM 1-43 for 5 minutes in an ice-cold chamber, on the microscope stage. For labeling after fixation, the organ of Corti was fixed for 60 minutes with 4% PFA and then imaged in HBSS without Ca^2+^ + 10 µM FM 1-43. For testing the effect of suramin (Sigma-Aldrich, Munich, Germany) on FM 1-43 uptake, organs of Corti were pre-incubated for 5 minutes in 100 µM suramin, then incubated for 1 minute in HBSS without Ca^2+^ + 5 µM FM 1-43 in presence of suramin, before washing and imaging.

### FM 1-43 labeling, photo-oxidation and EM processing

The apical turn of the organ of Corti was dissected and directly incubated in HBSS without Ca^2+^ for 5 minutes at 37°C. The organ was then subjected to one of the following experimental conditions: Resting, 60 seconds in HBSS without Ca^2+^ + 5 µM FM 1-43; Stimulation, 60 seconds in HBSS high K^+^ (KCl increased to 65.36 mM, NaCl reduced to 79.7 mM, plus 2 mM CaCl_2_) + 5 µM FM 1-43; Recovery, same as stimulated, followed by dye wash-off and incubation for 5 minutes in a constant flow of dye-free HBSS with Ca^2+^ (NaCl reduced to 139.7 mM plus CaCl_2_ 2 mM). All solutions were carbogen-charged and pre-warmed at 37°C before the experiments. After labeling the organs were fixed and washed in a large volume of 2.5% glutaraldehyde for 20 minutes on ice followed by 30 minutes at room temperature. Samples were quenched for 20 minutes in 100 mM NH_4_Cl, washed with PBS and incubated in di-amino-benzidine (DAB, 1.5 mg/ml in PBS) for 35 minutes at 4°C. Fresh DAB solution was applied. Photo-oxidation was performed as described before [30]. Briefly, organs of Corti were placed under an Olympus objective (20×, 0.5 NA, Olympus, Hamburg, Germany) installed in a Zeiss Axioskop 2 FS Plus microscope (Carl Zeiss AG, Oberkochen, Germany). By using a 100 W mercury lamp (Carl Zeiss), an excitation filter (470/40 nm) and a dichroic mirror (495 DCLP, AHF, Tübingen, Germany) the organ of Corti was illuminated in an area containing IHCs until a dark precipitate was formed (30–45 minutes). The photo-oxidized region was cut, washed with PBS, postfixed with 1% osmium tetroxide (Fluka, Sigma-Aldrich) for 1 hour and dehydrated in increasing concentrations of ethanol and propylene oxide (PO; Science Services, Munich, Germany). Samples were incubated in 50% epon resin (Science Services) in PO under continuous rotation for 12-18 hours, followed by incubation for 6 hours in 100% epon resin in open vials. Finally samples were embedded in fresh 100% epon resin and cured for 40 hours at 60°C. Hard epon blocks were cut into serial 100 nm thick sections using an ultra-microtome (Leica EM UC6, Leica). Sections were mounted on 0.5% formvar-coated copper grids (Plano GmbH, Wetzlar, Germany). Electron microscopy was performed using a Zeiss EM 902A microscope (Carl Zeiss) equipped with a 1024×1024 CCD detector (Proscan CCD HSS 512/1024; Proscan Electronic Systems). Images in Figs. 3, 4 and 6 were also acquired using an Orius SC1000A 1 camera (Gatan Inc., Pleasanton, CA), at 4008x2672 pixels, placed on a JEOL JEM1011 electron microscope (JEOL GmbH, Munich, Germany).

### Data analysis

For the quantification of photo-oxidized organelles in single electron microscopy sections, we performed at least 4 independent experiments for each condition. The photo-oxidized preparations were sectioned, and 3–5 sections were generated for each preparation. A series of images was taken for each IHC that could be identified in the 5 sections, including a few overview, low-zoom images, and up to 50 high-zoom images of areas throughout the cells. High-zoom images were acquired randomly in the four different regions of the IHCs: cuticular plate, top, nuclear, basal. The cuticular plate was typically photographed in its entirety. Regions of at least 8–12 µm^2^ were imaged for all other regions, in addition to the overview images. Afterwards, all images from photo-oxidized cells were analyzed, without favoring any cells over others.

For rendering IHC 3D reconstructions (Fig. 5A), organelles containing photo-oxidized product were first drawn by hand from electron micrographs using Adobe Photoshop CS3 (Adobe Systems Inc., San Jose, CA, USA) and then reconstructed with self-written routines in MatLab (The Mathworks Inc., Natick, MA, USA), as described in [30].

### Statistical analysis

All graphs show means ± SEM, unless otherwise indicated in the figure legends. The unpaired t-test was used for statistical analysis. Where indicated, we used a one-way ANOVA test, followed by a Bonferroni *post hoc* test. No blinding was used for data analysis, as each data set could be easily recognized by the experimenters.

## Supporting Information

Figure S1 **Reduction of FM 1-43 labeling by suramin is due to a direct interaction between these molecules.** Previous experiments [25] reported a reduction of FM 1-43 uptake in chicken hair cells in presence of suramin, an antagonist of the ATP-activated P2X receptors. We reproduced these experiments and found that suramin (containing negatively charged sulfonic groups) directly interacts with FM 1-43 (which is positively charged), and reduces its fluorescence, possibly by removing it from membranes. (**A**) Pre-incubation of organs of Corti with 100 µM Suramin drastically reduced labeling with FM 1-43 (5 µM, in presence of suramin). However, a 4-frame accumulation image shows that residual FM 1-43 fluorescence is still detectable throughout IHCs, with a distribution similar to that of control situations (Figs. 1 and 2), indicating that some dye still penetrates into the cells. Scale bar, 10 µm. (**B**) Suramin changes the color of FM 1-43 aqueous solutions, indicating that the two interact directly. (**C**) Suramin rapidly removes FM 1-43 from membranes. We added FM 1-43 onto pheochromocytoma (PC12) cells in culture (10 µM in PBS), and imaged their plasma membranes within seconds of FM 1-43 application (left panel). Addition of 100 µM suramin, in presence of FM dye, reduced the fluorescence drastically (right panel and **D**), most likely through a direct interaction with the FM dye. Endocytosed organelles and inter-cellular spaces in large PC12 cell clumps, where suramin could not penetrate, remained bright. Scale bar, 40 µm. (**D**) Analysis of the effect of suramin on FM 1-43 fluorescence. 360 PC12 cells were analyzed, in two independent experiments. The FM 1-43 fluorescence was measured both before and after suramin application. The difference is significant (t-test, *P*<0.001). (**E**) Effects of suramin on the excitation and emission spectra of FM 1-43, in aqueous solution. Note that suramin binding changes the excitation spectrum of FM 1-43. The spectra were acquired using a FluoroMax spectrophotometer, using 2 nm increments across the spectra, and 1 second integration time for each measurement.
(TIF)

Figure S2 **Paraformaldehyde fixation creates pores on the plasma membrane, through which FM 1-43 diffuses inside the cells.** Fibroblast cells in culture (COS7) were incubated with FM 1-43 (5 µM in Tyrode buffer; 124 mM NaCl, 5 mM KCl, 2 mM CaCl_2_, 1 mM MgCl_2_, 30 mM glucose, 25 mM HEPES, pH 7.4), before and after fixation with 4% paraformaldehyde. Note that only the plasma membrane is labeled in living cells. The cellular organelles are revealed after fixation, due to the entry of the dye through pores in the membrane. The cells were imaged in identical conditions, and the images are scaled similarly. Scale bar, 20 µm.
(TIF)

Figure S3 **FM dyes and their analogs label IHCs at low temperature.** (**A**) Different styryl dyes and DCF (10 µM) were applied to organs of Corti placed in a cold imaging chamber (on ice, 2-4°C). IHCs were strongly labeled, which confirms the endocytosis-independent entry of the dyes into these cells. (**B**) Similar experiment for the membrane-binding dye Di-2-ANEPEQ. The quantification of these experiments can be found in Fig. 2.
(TIF)

Figure S4 **HRP does not label IHC organelles as efficiently as FM dyes.** Organs of Corti were incubated with HRP (20 minutes), and were photo-oxidized under the same conditions as for FM 1-43 (stimulated preparations, as in Fig. 4B). The diffusion of HRP into the tissue (between cells) was poor, and therefore did not result in IHC labeling. Scale bar, 500 nm. This image is typical for three independent experiments.
(TIF)

Figure S5 **Tubulo-cisternal organelles from IHCs.** (**A**) Three-dimensional reconstruction of a tubulo-cisternal organelle from a resting cell. Scale bar, 200 nm. (**B**) Nine consecutive sections containing labeled organelles are shown, from a cell imaged at rest. One organelle of interest is indicated by the dashed red traces. Mitochondria are indicated by white asterisks. Scale bar, 200 nm.
(TIF)
