# Supplementary figures and images for: FM Dye Photo-Oxidation as a Tool for Monitoring Membrane Recycling in Inner Hair Cells
Source: PLoS One. 2014 Feb 5;9(2):e88353. doi: 10.1371/journal.pone.0088353 (PMC3914975; doi:10.1371/journal.pone.0088353)

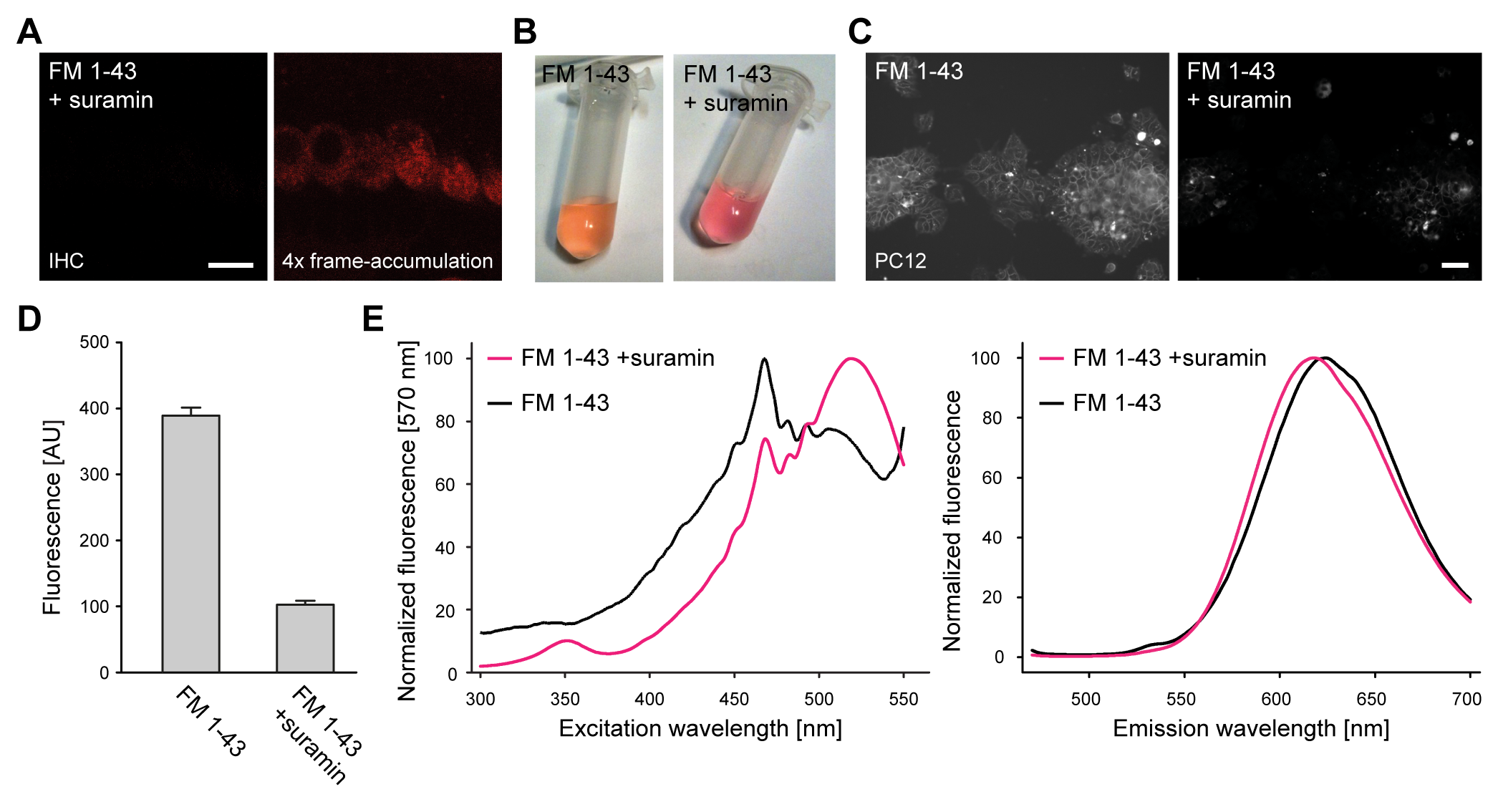

Supplement: Figure S1 — Reduction of FM 1-43 labeling by suramin is due to a direct interaction between these molecules. Previous experiments [25] reported a reduction of FM 1-43 uptake in chicken hair cells in presence of suramin, an antagonist of the ATP-activated P2X receptors. We reproduced these experiments and found that suramin (containing negatively charged sulfonic groups) directly interacts with FM 1-43 (which is positively charged), and reduces its fluorescence, possibly by removing it from membranes. (A) Pre-incubation of organs of Corti with 100 µM Suramin drastically reduced labeling with FM 1-43 (5 µM, in presence of suramin). However, a 4-frame accumulation image shows that residual FM 1-43 fluorescence is still detectable throughout IHCs, with a distribution similar to that of control situations (Figs. 1 and 2), indicating that some dye still penetrates into the cells. Scale bar, 10 µm. (B) Suramin changes the color of FM 1-43 aqueous solutions, indicating that the two interact directly. (C) Suramin rapidly removes FM 1-43 from membranes. We added FM 1-43 onto pheochromocytoma (PC12) cells in culture (10 µM in PBS), and imaged their plasma membranes within seconds of FM 1-43 application (left panel). Addition of 100 µM suramin, in presence of FM dye, reduced the fluorescence drastically (right panel and D), most likely through a direct interaction with the FM dye. Endocytosed organelles and inter-cellular spaces in large PC12 cell clumps, where suramin could not penetrate, remained bright. Scale bar, 40 µm. (D) Analysis of the effect of suramin on FM 1-43 fluorescence. 360 PC12 cells were analyzed, in two independent experiments. The FM 1-43 fluorescence was measured both before and after suramin application. The difference is significant (t-test, P<0.001). (E) Effects of suramin on the excitation and emission spectra of FM 1-43, in aqueous solution. Note that suramin binding changes the excitation spectrum of FM 1-43. The spectra were acquired using a FluoroMax spectropho [file pone.0088353.s001.tif]

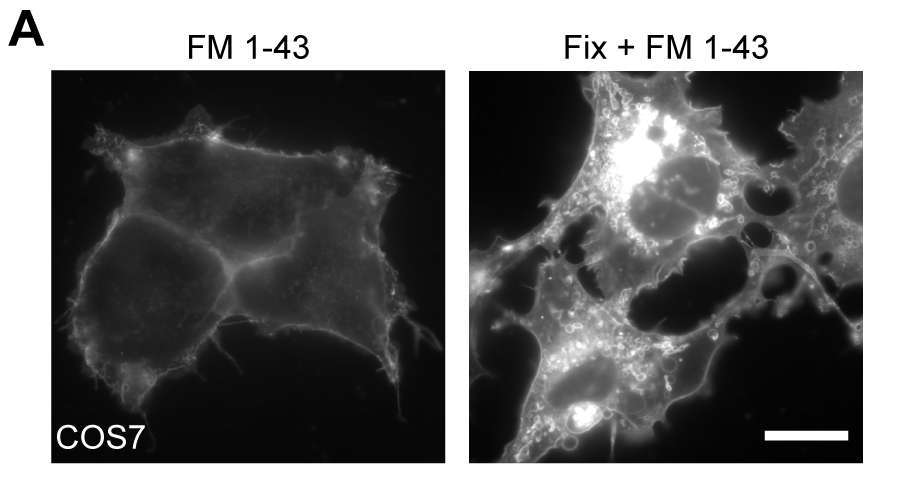

Supplement: Figure S2 — Paraformaldehyde fixation creates pores on the plasma membrane, through which FM 1-43 diffuses inside the cells. Fibroblast cells in culture (COS7) were incubated with FM 1-43 (5 µM in Tyrode buffer; 124 mM NaCl, 5 mM KCl, 2 mM CaCl2, 1 mM MgCl2, 30 mM glucose, 25 mM HEPES, pH 7.4), before and after fixation with 4% paraformaldehyde. Note that only the plasma membrane is labeled in living cells. The cellular organelles are revealed after fixation, due to the entry of the dye through pores in the membrane. The cells were imaged in identical conditions, and the images are scaled similarly. Scale bar, 20 µm. (TIF) [file pone.0088353.s002.tif]

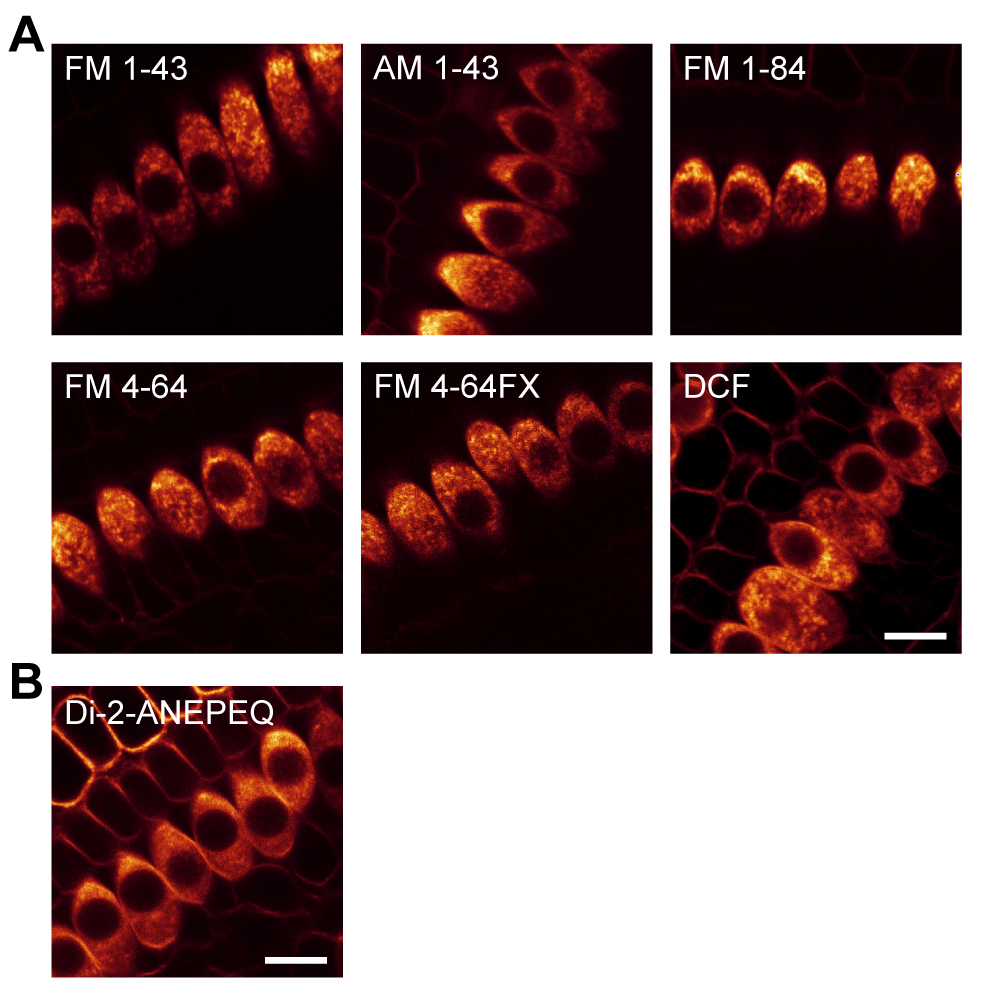

Supplement: Figure S3 — FM dyes and their analogs label IHCs at low temperature. (A) Different styryl dyes and DCF (10 µM) were applied to organs of Corti placed in a cold imaging chamber (on ice, 2-4°C). IHCs were strongly labeled, which confirms the endocytosis-independent entry of the dyes into these cells. (B) Similar experiment for the membrane-binding dye Di-2-ANEPEQ. The quantification of these experiments can be found in Fig. 2. (TIF) [file pone.0088353.s003.tif]

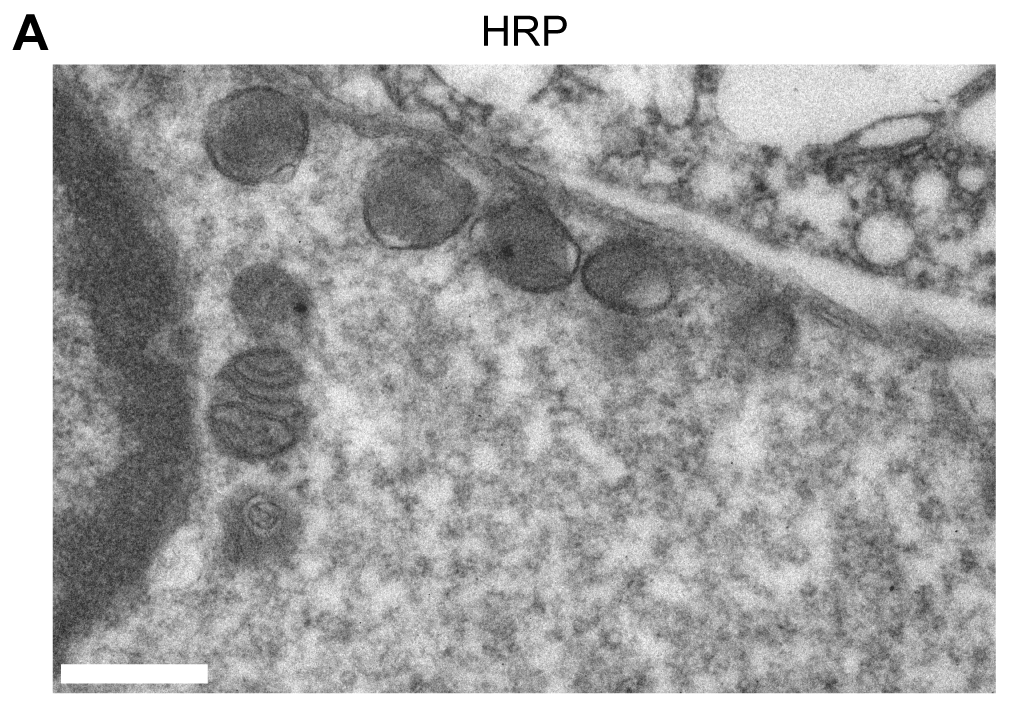

Supplement: Figure S4 — HRP does not label IHC organelles as efficiently as FM dyes. Organs of Corti were incubated with HRP (20 minutes), and were photo-oxidized under the same conditions as for FM 1-43 (stimulated preparations, as in Fig. 4B). The diffusion of HRP into the tissue (between cells) was poor, and therefore did not result in IHC labeling. Scale bar, 500 nm. This image is typical for three independent experiments. (TIF) [file pone.0088353.s004.tif]

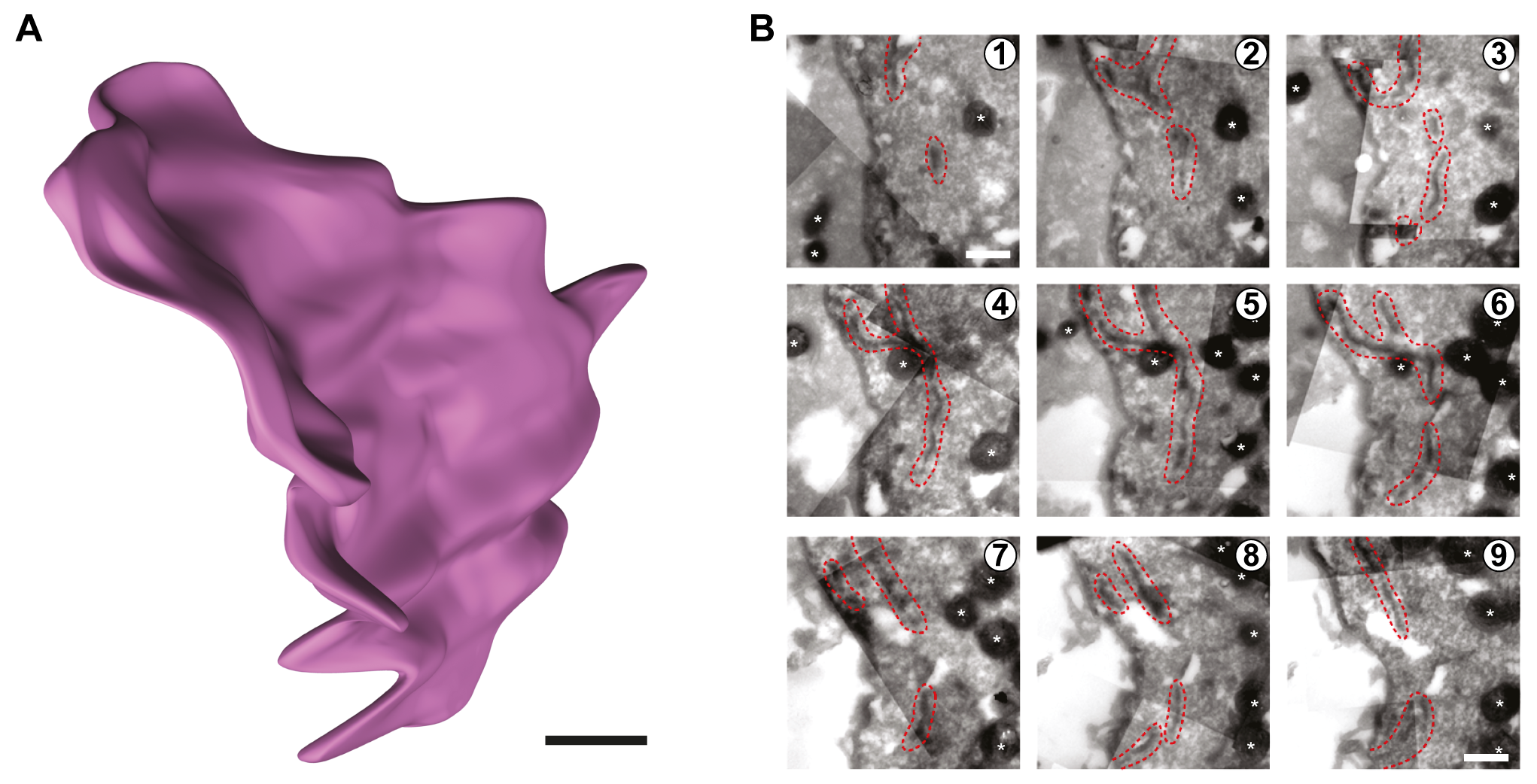

Supplement: Figure S5 — Tubulo-cisternal organelles from IHCs. (A) Three-dimensional reconstruction of a tubulo-cisternal organelle from a resting cell. Scale bar, 200 nm. (B) Nine consecutive sections containing labeled organelles are shown, from a cell imaged at rest. One organelle of interest is indicated by the dashed red traces. Mitochondria are indicated by white asterisks. Scale bar, 200 nm. (TIF) [file pone.0088353.s005.tif]
